# Organic-skinned inorganic nanoparticles: surface-confined polymerization of 6-(3-thienyl)hexanoic acid bound to nanocrystalline TiO_2_

**DOI:** 10.1186/1556-276X-6-521

**Published:** 2011-09-02

**Authors:** Viswanathan S Saji, Yimhyun Jo, Hoi Ri Moon, Yongseok Jun, Hyun-Kon Song

**Affiliations:** 1 i-School of Green Energy, UNIST, Ulsan 689-798, South Korea

**Keywords:** surface-bound polymerization, nanocrystalline TiO_2_, thiophenes, FeCl_3_

## Abstract

There are many practical difficulties in direct adsorption of polymers onto nanocrystalline inorganic oxide surface such as Al_2_O_3 _and TiO_2 _mainly due to the insolubility of polymers in solvents or polymer agglomeration during adsorption process. As an alternative approach to the direct polymer adsorption, we propose surface-bound polymerization of pre-adsorbed monomers. 6-(3-Thienyl)hexanoic acid (THA) was used as a monomer for poly[3-(5-carboxypentyl)thiophene-2,5-diyl] (PTHA). PTHA-coated nanocrystalline TiO_2_/FTO glass electrodes were prepared by immersing THA-adsorbed electrodes in FeCl_3 _oxidant solution. Characterization by ultraviolet/visible/infrared spectroscopy and thermal analysis showed that the monolayer of regiorandom-structured PTHA was successfully formed from intermolecular bonding between neighbored THA surface-bound to TiO_2_. The anchoring functional groups (-COOH) of the surface-crawling PTHA were completely utilized for strong bonding to the surface of TiO_2_.

## Introduction

Conducting polymers have attracted widespread academic and industrial research interest in the last two decades because of their potential applications in various fields such as light-emitting diodes, electrochromic devices, photovoltaic cells, anti-corrosion coatings, sensors, batteries, and supercapacitors [1-3]. Polythiophenes are one of the most widely studied conjugated conducting polymers due to their electrical properties, stability in doped and undoped states, nonlinear optical properties, and highly reversible redox switching [4,5]. Thiophene derivatives can be polymerized chemically, photochemically, or electrochemically to the corresponding oligothiophenes or polythiophenes [6-8]. However, poor processability of polythiophenes caused by their low solubility in solvents has impeded their practical applications. Even after grafting flexible hydrocarbon chains onto the polymer backbone, their solubility in most of organic solvents and water is too low. Despite the intensive research efforts for developing highly soluble and easily processable polythiophenes, yields of soluble polythiophenes were extremely low and/or synthetic processes demanded high costs and use of toxic solvents [9,10].

Oligothiophenes and polythiophenes have strong potentials in solar cell applications, functioning as a donor material in bulk heterojunction solar cells, as a hole-transporting layer in solid-state dye-sensitized solar cells (DSSCs) and as a light-absorbing species that injects electrons into the conduction band of n-type semiconductor (e.g., TiO_2_) in DSSCs [11,12]. Especially in the third cases, infiltrating sufficient amount of polymer into porous void of the nanostructured metal oxide electrodes is critical in obtaining high efficiency of polymeric-dye-based DSSCs. The cell performances are limited by the poor penetration of polymers into the porous nanocrystalline TiO_2 _network. Also, polymer aggregation within a void of porous electrodes can cause problems.

Instead of infiltrating pre-synthesized polymers, *in situ *formation of oligothiophenes or polythiophenes within nanostructured architectures would be one of the possible alternative ways to overcome the obstacles (low solubility, difficult infiltration into porous structure, and polymer aggregation). Several different polymerization strategies can be considered as the *in situ *formation of polymer. Electropolymerization of monomers would enable the *in situ *polymerization only if the substrate in which polymer is formed were conductive. High vacuum techniques including laser-induced vapor deposition; plasma polymerization; and × ray-, electron-, and ion-induced synthesis result in fragmentation of the monomer structure leading to defective incorporation into a target substrate [13]. Photochemical and chemical polymerization [14,15] in a solution phase led to a successful deposition of polythiophenes onto nanostructured TiO_2 _electrodes. Zhang et al. [14] grafted poly(3-hexylthiophene) or P3HT chemically on a modified surface of TiO_2 _nanotubes. The polymerization was initiated from the monolayered 3HT-containing molecules covalently bound to TiO_2_. Fe^3+ ^was used as an oxidizing agent to proceed polymerization in presence of the monomer 3HT. Tepavcevic et al. [15] polymerized 2,5-diiodothiophene (DIT) as monomer precursor on the surface of TiO_2 _nanotubes photochemically by ultraviolet irradiation. A thienyl radical and iodine atoms dissociated from DIT by UV absorption were preferentially adsorbed on TiO_2 _surface, forming initiation sites for polymerization. The reason for the surface specificity is that TiO_2 _serves as the primary conduit for transferring light energy. The photochemical and chemical polymerization can be classified as the surface-initiated polymerization in which direction of polymer growth was out of plane of target substrate.

**Figure 1:**
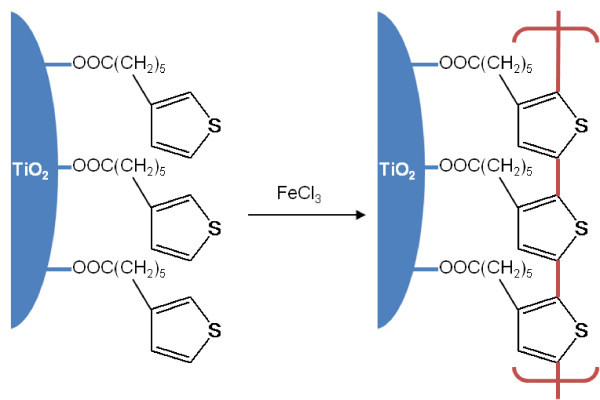
**Surface-bound polymerization of THA to PTHA on surface of a TiO_2 _nanocrystallite**. The monomer THA was strong bonded to TiO_2 _surface via -COOH. FeCl_3 _was used as an oxidizing agent to polymerize the surface-bound THA to its corresponding polymer PTHA.

In this context, it would be interesting to investigate whether polymerization is possible not between adsorbed monomers and free monomers in a solvent but between adsorbed ones. The surface-bound polymerization would lead to polymeric growth in a direction parallel to surface, forming a consecutively side-by-side bonded monolayer (Figure 1). In this work, therefore, we investigated a model system as the representative surface-bound polymerization. Carboxyl-functionalized thiophene monomer was adsorbed onto surface of nanocrystalline TiO_2 _electrodes. The -COOH groups facilitates strong linking of monomers onto TiO_2_. After removing extra free or loosely bound monomers from the TiO_2 _surface, the surface-bound monomers were polymerized in absence of free monomers in solution by using Fe^3+ ^as an oxidant.

## Experimental

A commercial paste including TiO_2 _nanoparticles (T20, Solaronix, Switzerland) was coated on fluorine-doped tin oxide glass plates (SnO_2_:F, FTO) by a doctor blade and then sintered at 450°C for 30 min in a muffle furnace. The thickness of sintered films was estimated at approximately 10 μm by a surface profilometer.

A typical procedure of surface-bound polymerization is described as follows. The TiO_2_-coated electrodes were heated at 120°C for 10 min. After being cooled down to a specific temperature between room temperature and 80°C, the electrodes were immersed in a 20 mM monomer solution in acetonitrile for 24 h. 6-(3-Thienyl)hexanoic acid (THA, #4132, Rieke Metals, USA) was used as the monomer that is adsorbed on the immersion step. After the THA-adsorbed electrodes were rinsed thoroughly by acetonitrile and dried in air, they were dipped into a 10 mM FeCl_3 _solution in acetonitrile and kept stagnant during a specific time period. Then, the resultant polymer-adsorbed electrodes were washed repeatedly in copious amount of 1:1 mixture of methanol and ethanol to remove loosely bound species including polymers and ferric or ferrous ions.

As a control to the polymer-adsorbed TiO_2 _electrodes obtained by polymerizing the surface-bound THA, poly[3-(5-carboxypentyl)thiophene-2,5-diyl] (PTHA, Rieke 4032) was directly adsorbed on the same TiO_2 _electrodes. TiO_2 _electrodes were immersed in a 20 mM solution of PTHA in acetonitrile for 24 h. The immersion temperature was fixed at 80°C since the solubility of PTHA in acetonitrile is very low at room temperature. After the polymer adsorption, electrodes were repeatedly washed in acetonitrile to remove any loosely bound species.

The PTHA-adsorbed electrodes prepared from the surface-bound polymerization or direct adsorption were characterized by ultraviolet-visible spectroscopy (UV-vis, 2401PC, Shimadzu, Japan), Fourier-transformed infrared spectroscopy (FTIR, Varian 670, Varian, USA), and thermogravimetric analysis (TGA, TA SDT Q600; with a nitrogen atmosphere, TA instruments, USA).

## Results and discussion

**Figure 2:**
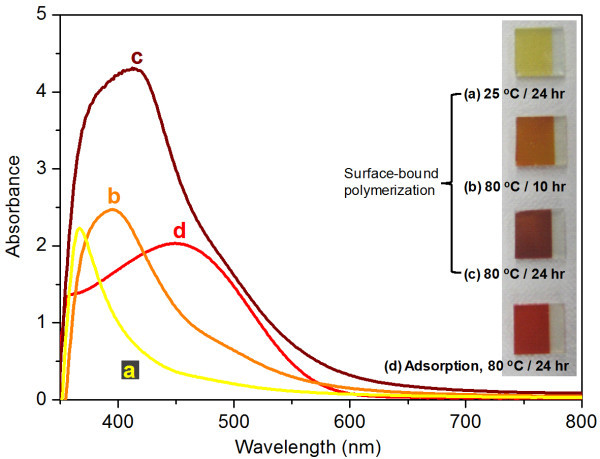
**UV-vis spectra of PTHA-coated TiO_2 _electrodes**. (**a**, **b**, **c**) PTHA prepared by surface-bound polymerization with various oxidizing conditions: dipping in FeCl_3 _(a) at room temperature for 24 h, (b) at 80°C for 10 h and (c) at 80°C for 24 h. (**d**) PTHA prepared via direct polymer adsorption by dipping TiO_2 _electrodes in PTHA solution at 80°C for 24 h. (Inset) Photograph of PTHA-coated TiO_2 _electrodes.

Growth of PTHA or oligo-THA via surface-bound polymerization was traced by UV-vis absorption. Figure 2 shows the absorption spectra of PTHA or oligo-THA obtained by polymerizing surface-bound THA on TiO_2 _electrodes at different conditions of polymerization temperature and time. For a comparison, the spectrum of PTHA adsorbed on the same porous TiO_2 _electrode at 80°C for 1 day is also shown. A bare TiO_2 _electrode was employed as the reference. Typically, polythiophenes exhibit absorption maximum around 500 nm with an extended absorption tail reaching up to 650 nm [16]. The absorption peak of oligomer or polymer obtained by surface-bound polymerization was observed at ~350 nm (Figure 2a) at room temperature. Its long tail extending up to 600 nm indicates some degree of oligomer/polymer formation. By increasing polymerization temperature (even with a shorter reaction time), the absorption peak gradually shifted to longer wavelength region or red color region (from 350 nm (a) through 400 nm (b) to 415 nm (c) in Figure 2). Simultaneously, the color of electrodes changed apparently from yellow through orange to dark red (the inset in Figure 2). The broad absorption in the range of 350 to 700 nm with strong absorbance (Figure 2c) guarantees significant formation of oligo/polythiophenes. As absorption is directly related to the polymer π conjugation length, it can be presumed that significant oligomerization or polymerization proceeded at higher temperature and longer reaction time. This is attributed to enhanced mobility of the adsorbed monomers and accelerated oxidation kinetics of monomers at higher temperatures which might have facilitated polymerization of adjacent thiophenes in the monolayer.

For comparison, the control sample obtained by polymer adsorption (Figure 2d) shows higher peak wavelength at 450 nm with lower intensity (versus Figure 2c), demonstrating more bright red color. Considering that the used PTHA for polymer adsorption is highly regioregular (98.5% or higher), the blue-shifted spectrum for surface-bound polymerization is related to a structure-less monolayer of PTHA of regiorandom geometry in nature with shorter conjugation lengths [15]. In the conventional FeCl_3_-based polymerization of substituted thiophenes, polymerization happens through either 2- or 5-position of adjacent five-membered monomers. When a monomer is incorporated in a growing polymer chain, it can be added either with its head (2-position) or tail (5-position), resulting in three different possible couplings [17]. The propagation is believed to be initiated by a thiophene *radical cation*. Then, the propagation proceeds through a *carbocation *since polymer chain cannot be neutral under the strong oxidizing conditions [18]. In electrochemical polymerization, on the other hand, the oxidation of monomers produces a radical cation which can then be coupled with a next radical cation to form a di-cation dimer. The process repeats and hence the polymer chain grows [19]. Tepavcevic et al. reported that UV irradiation caused the C-I bond of adsorbed monomers (2,5-diiodothiophene) to be selectively photodissociated and then produced monomer radicals with intact π ring structure that further coupled to oligothiophenes/polythiophenes molecules [15]. In the present case, the functional group of PTHA is strongly bonded to the TiO_2 _surface. As soon as the electrodes were dipped in the oxidant solution, a radical cation is formed in each monomer. Due to the geometric restriction of surface-bound configuration, propagation proceeds between adjacent adsorbed monomers. Also, with the same reason, regiorandom structure is preferred with a limited degree of polymerization.

**Figure 3:**
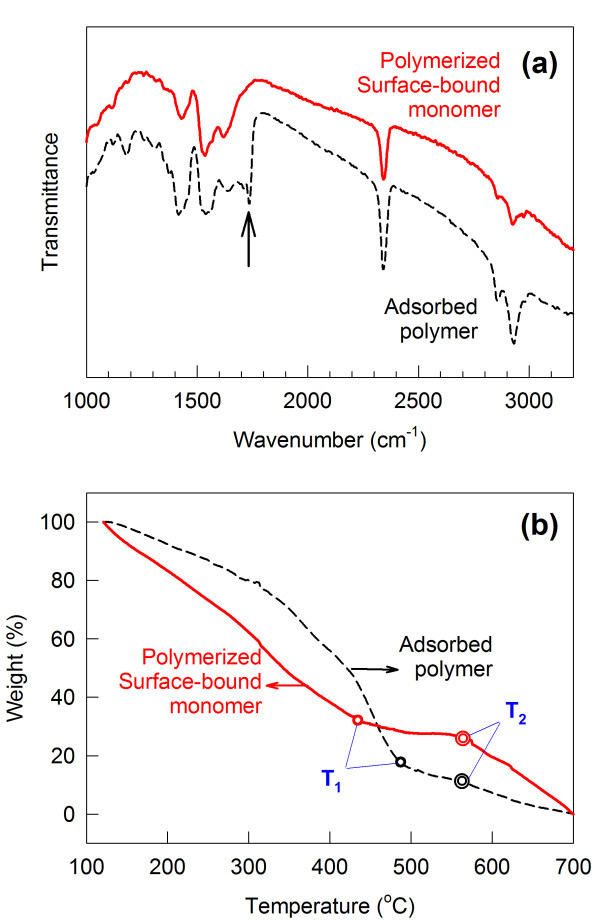
**FTIR spectra and thermograms of PTHA-coated TiO_2 _electrodes**. (**a**) FTIR spectra and (**b**) thermograms of PTHA-coated TiO_2 _electrodes for surface-bound polymerization versus direct polymer adsorption. An inert atmosphere was kept at 20°C min^-1 ^for TGA.

FTIR spectra were compared between PTHAs prepared by surface-bound polymerization and direct adsorption on TiO_2 _(Figure 3a). Qualitatively similar spectra were obtained from both samples, consistent with that of polythiophenes [20]. The surface-bound polymerization showed lower intensities of the peaks corresponding to aliphatic and aromatic C-H stretching (2,850 and 2,930 cm^-1^), compared with polymer adsorption. It indicates that smaller amount of PTHA is obtained or degree of polymerization is limited with surface-bound polymerization. This is easily understandable since the amount of monomers and the intermolecular collision between surface-bound monomers cannot help being limited. Both of PTHA have the similar intensity of peaks centered at 1,380 and 1,630 cm^-1 ^ascribed to the symmetric and anti-symmetric stretch modes of the carboxylate group [21]. Monomer molecules (THA) for surface-bound polymerization would be adsorbed at full coverage over TiO_2 _if the whole adsorption sites of TiO_2 _surface are occupied by polymer PTHA for polymer adsorption as the control. However, the peak at 1,720 cm^-1 ^attributed to free carboxylic acid group (indicated by arrow in Figure 3a) is observed only with PTHA prepared by polymer adsorption. There exist free -COOH groups in the polymer backbone which are not strongly bound to TiO_2 _surface. The clear absence of the peak with surface-bound polymerization supports all of the carboxylate functional group is completely used for bonding to TiO_2 _surface. In other words, all of the -COOH groups in a polymer backbone does not necessarily get involved in adsorption process of direct polymer adsorption.

To support conclusions from FTIR spectra, mass change was investigated with temperature by TGA (Figure 3b). Samples were obtained by scratching PTHA-coated TiO_2 _electrodes prepared by surface-bound polymerization and polymer adsorption. The weight percent (*m*_%_) was calculated by: *m*_% _= (*m *- *m*_700_)/(*m*_110 _- *m*_700_) with *m *= mass at a certain temperature, m_700 _and m_110 _= mass at 700°C and 110°C. Since TiO_2 _is stable within the temperature range examined, PTHA is wholly responsible for the weight loss. Three regions of degradation processes were clearly shown for both of PTHA [22,23]:

1. Small molecule decomposition region (up to *T*_1 _indicated by circle in Figure 3b, T_1 _= 430°C for surface-bound polymerization and 490°C for polymer adsorption): ascribed to loss of doped molecules or pendanted molecular structure including Cl^- ^as a dopant, functional groups, and a small fraction of thiophene;

2. Thermally stable region (between *T*_1 _and *T*_2_);

3. Polymer degradation region (from *T*_2 _indicated by double circle in Figure 3b): oxidative degradation of polymer backbone.

Even if characteristic polymer decomposition looks similar in both cases at the first look, a closer analysis of the thermograms leads to the conclusion that is obtained above from FTIR: smaller amount of PTHA or lower degree of polymerization with surface-bound polymerization. Lower *T*_1 _indicates the smaller amount of PTHA formed on surface while the abrupt decrease of mass after *T*_2 _in the region (3) is due to the low degree of polymerization.

## Conclusions

We showed that specifically surface-crawling polymer can be developed by polymerizing its corresponding monomers surface-bound to metal oxide nanoparticles. As a model of the organic/inorganic hybrid system, TiO_2 _and THA were chosen as the inorganic nano-substrate and the organic monomer that will be polymerized into PTHA, respectively. All of the anchoring functional groups (-COOH) were completely used for connecting polymer backbone to the surface of TiO_2_, while free carboxylates not participating in bonding were observed with direct polymer adsorption on TiO_2_. Degree of oligomerization/polymerization or the total amount of PTHA was limited by the geometric restriction of the surface-bound THA. Although the polymers obtained by this method may have lower regioregularity and π conjugation, the specifically surface-confined polymerization would be of a reference methodology for basic studies of completely surface-bonded polymer films and for developing hybrid solar cells and organic electronics.

## Competing interests

The authors declare that they have no competing interests.

## Authors' contributions

VSS proposed the original idea, carried out most of experiments including synthesis and analysis and wrote the first draft of manuscript. YJ analyzed material properties. HRM and YJ detailed the original idea and modified the first draft of manuscript. HKS designed and coordinated the whole work and finalized the manuscript. All authors read and approved the final manuscript.
